# Molecular Mechanism Underlying the Action of *Zona-pellucida* Glycoproteins on Mouse Sperm

**DOI:** 10.3389/fcell.2020.572735

**Published:** 2020-08-31

**Authors:** Melanie Balbach, Hussein Hamzeh, Jan F. Jikeli, Christoph Brenker, Christian Schiffer, Jan N. Hansen, Pia Neugebauer, Christian Trötschel, Luca Jovine, Ling Han, Harvey M. Florman, U. Benjamin Kaupp, Timo Strünker, Dagmar Wachten

**Affiliations:** ^1^Center of Advanced European Studies and Research, Department of Molecular Sensory Systems, Bonn, Germany; ^2^Institute of Innate Immunity, Department of Biophysical Imaging, Medical Faculty, University of Bonn, Bonn, Germany; ^3^Center of Reproductive Medicine and Andrology, University Hospital Münster, University of Münster, Münster, Germany; ^4^Biochemie der Pflanzen, Ruhr-Universität Bochum, Bochum, Germany; ^5^Department of Biosciences and Nutrition, Karolinska Institutet, Solna, Sweden; ^6^Department of Obstetrics and Gynecology, University of Massachusetts Medical School Worcester, Worcester, MA, United States; ^7^Life and Medical Sciences Institute, University of Bonn, Bonn, Germany

**Keywords:** *zona pellucida*, mouse sperm, calcium, sperm motility, sodium-proton exchange

## Abstract

Mammalian oocytes are enveloped by the zona pellucida (ZP), an extracellular matrix of glycoproteins. In sperm, stimulation with ZP proteins evokes a rapid Ca^2+^ influx via the sperm-specific, pH-sensitive Ca^2+^ channel CatSper. However, the physiological role and molecular mechanisms underlying ZP-dependent activation of CatSper are unknown. Here, we delineate the sequence of ZP-signaling events in mouse sperm. We show that ZP proteins evoke a rapid intracellular pH_*i*_ increase that rests predominantly on Na^+^/H^+^ exchange by NHA1 and requires cAMP synthesis by the soluble adenylyl cyclase sAC as well as a sufficiently negative membrane potential set by the spem-specific K^+^ channel Slo3. The alkaline-activated CatSper channel translates the ZP-induced pH_*i*_ increase into a Ca^2+^ response. Our findings reveal the molecular components underlying ZP action on mouse sperm, opening up new avenues for understanding the basic principles of sperm function and, thereby, mammalian fertilization.

## Introduction

The *zona pellucida* (ZP) serves as an important check-point during fertilization, allowing sperm from the same (homologous) species to penetrate the ZP and fuse with the oocyte, while preventing the penetration of sperm from a different (heterologous) species. Fertilization requires sperm capacitation, a maturation process occurring in the female genital tract (Chang, 1951; Austin, 1952; Braden et al., 1954; Avella et al., 2014; Fahrenkamp et al., 2020). After fertilization, the ZP becomes impenetrable also to homologous sperm, avoiding the fertilization of the oocyte by more than one sperm cell, called polyspermy (Avella et al., 2013; Gupta, 2015). Moreover, *in vitro*, binding of sperm to the ZP changes swimming behavior (Katz and Yanagimachi, 1981; Baltz et al., 1988; Drobnis et al., 1988; Sumigama et al., 2015), and stimulation of sperm with solubilized ZP proteins evokes acrosomal exocytosis (Florman and Storey, 1982; Bleil and Wassarman, 1983; Baltz et al., 1988; Cross et al., 1988; Florman et al., 1989; Arnoult et al., 1996a, b), which is also evoked upon binding of sperm to isolated, intact ZPs (Jin et al., 2011; Sumigama et al., 2015). The significance and role of the ZP-induced acrosome reaction for fertilization are, however, unclear and might vary between mammalian species (Jin et al., 2011; La Spina et al., 2016; Muro et al., 2016; Hirohashi and Yanagimachi, 2018). Furthermore, the signaling pathways underlying the action of ZP proteins on sperm are only ill-defined (Florman et al., 2008).

Swimming behavior and acrosomal exocytosis are controlled by changes in the intracellular Ca^2+^ concentration ([Ca^2+^]_*i*_) (Ho and Suarez, 2001; Eisenbach and Giojalas, 2006; Florman et al., 2008; Kaupp et al., 2008; Publicover et al., 2008). In mouse sperm, ZP proteins evoke a Ca^2+^influx (Arnoult et al., 1996a, 1999; Florman et al., 2008) via the sperm-specific Ca^2+^ channel CatSper (Xia and Ren, 2009). How ZP proteins activate CatSper is not known. In general, CatSper is activated upon depolarization of the membrane potential (V_*m*_) and alkalization of the intracellular pH (pH_*i*_) (Kirichok et al., 2006; Lishko and Kirichok, 2010; Lishko et al., 2011; Strünker et al., 2011; Seifert et al., 2015). ZP proteins evoke a pH_*i*_ increase, which might stimulate alkaline-evoked Ca^2+^ influx via CatSper (Arnoult et al., 1996b, 1999), The sperm-specific Na^+^/H^+^ exchanger sNHE (Slc9c1) (Wang et al., 2003) has been proposed to mediate the pH_*i*_ increase (Chavez et al., 2014). Finally, ZP-evoked Ca^2+^ influx in mouse sperm requires a sufficiently negative membrane potential (V_*m*_), set by the sperm-specific K^+^ channel Slo3 (Kcnu1, Table 1) (Chavez et al., 2014). However, the mechanism underlying the V_*m*_-control of the ZP action has remained enigmatic.

**TABLE 1:** Official nomenclature for genes and proteins used in this study.

	Mouse	Official protein name
**Gene**	*Catsper*	Cation channel sperm-associated protein 1
**Protein**	CATSPER	
**Alias**	CatSper	
**Gene**	*Slc9b1*	Sodium/hydrogen exchanger 9B1
**Protein**	Slc9b1	
**Alias**	NHA1	
**Gene**	*Slc9c1*	Sodium/hydrogen exchanger 10
**Protein**	Slc9c1	
**Alias**	sNHE	
**Gene**	*Kcnu1*	Potassium channel subfamily U member 1
**Protein**	Kcnu1	
**Alias**	Slo3	
**Gene**	Adcy10	Adenylyl cyclase type 10
**Protein**	*Adcy10*	
**Alias**	sAC	
**Gene**	*Lrrc52*	Leucine-rich repeat-containing protein 52
**Protein**	Lrrc52	

Here, we study the action of solubilized ZP proteins on mouse sperm. We show that the ZP-induced pH_*i*_ increase is required to evoke Ca^2+^ influx via CatSper. The pH_*i*_ increase is abolished upon depolarization, which underlies the V_*m*_-control of CatSper activation by ZP proteins. Moreover, we show that the ZP-induced pH_*i*_ increase rests on Na^+^/H^+^ exchange by NHA1, but not by sNHE, and requires cAMP synthesis by the soluble adenylyl cyclase sAC. Altogether, our findings answer long-standing questions about the molecular mechanisms underlying ZP action on mouse sperm.

## Results

### ZP-Induced Ca^2+^- and pH_*i*_-Signaling Events in Mouse Sperm

**FIGURE 1:**
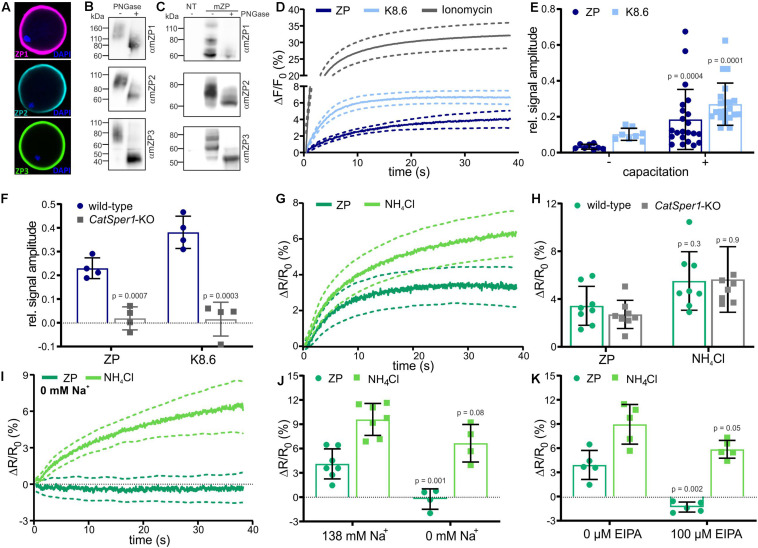
Changes in pH_*i*_ and [Ca^2+^]_*i*_ stimulated by solubilized *zona pellucida* (ZP) glycoproteins in mouse sperm. **(A)** Mouse oocytes labeled with antibodies directed against mZP1 (purple), mZP2 (cyan), and mZP3 (green); the DNA was labeled using DAPI (blue). **(B)** Western blot of solubilized ZP glycoproteins before (–) and after (+) PNGase-F treatment. The blots were probed with ZP isoform-specific antibodies. **(C)** Western blot of heterologously-expressed mouse ZP glycoproteins before (–) and after (+) PNG-F treatment, probed with mZP isoform-specific antibodies; NT: non-transfected cells. **(D)** Ca^2+^ responses in populations of sperm evoked by mixing with 1 ZP/μl, K8.6 buffer, or 2 μM ionomycin; shown are the averages (solid lines, *n* = 7) and the 95% confidence interval (dashed lines). Shown is the percent change in fluorescence (ΔF) with respect to the mean of the first three data points recorded immediately after mixing (F_0_). The control ΔF/F_0_ signal observed upon mixing with buffer (control) was subtracted from K8 6-, ZP-, or ionomycin-induced signals, setting the control-signal level to ΔF/F (%) = 0. **(E)** Relative amplitude of the Ca^2+^ responses evoked by mixing with 1 ZP/μl or K8.6 (mean ± SD of the average of the last three data points, *n*≥7) in non-capacitated (–) and capacitated (+) sperm, normalized to the ionomycin-evoked Ca^2+^ responses. **(F)** Relative amplitude of Ca^2+^ responses in wild-type and *Catsper1*-KO sperm (*n* = 4). **(G)** pH_*i*_ responses evoked by mixing with 1 ZP/μl or 10 mM NH_4_Cl in mouse sperm populations; shown are averages (solid lines, *n* = 7) and the 95% confidence interval (dashed lines). Shown is the percent change in fluorescence ratio (ΔR) with respect to the mean of the first three data points recorded immediately after mixing (R_0_). The control ΔR/R_0_ signal observed upon mixing with buffer (control) was subtracted, setting the control-signal level to ΔR/R_0_ (%) = 0. **(H)** Amplitude of pH_*i*_ responses (mean ± SD of the average of the last three data points, *n*≥ 7) evoked by mixing with 1 ZP/μl or 10 mM NH_4_Cl in wild-type and *Catsper1*-KO sperm (*n* = 4). **(I)** pH_*i*_ responses in sperm bathed in 0 mM Na^+^ buffer (*n* = 4). **(J)** Amplitude of pH_*i*_ responses at 138 or 0 mM extracellular Na^+^ (mean ± SD, *n* = 4). **(K)** Amplitude of pH_*i*_ responses in the absence (–) or presence (+) of 100 μM EIPA (mean ± SD, *n* = 4). Statistical significance between two groups was determined using two-tailed, unpaired *t*-test with Welch’s correction; *p*-values are indicated.

We studied the action of solubilized ZP proteins on mouse sperm. In mice, the ZP consists of three glycoproteins (mZP1-3). In line with previous studies (Avella et al., 2014), staining of isolated oocytes with antibodies against mZP1, mZP2, and mZP3 labeled the ZP surrounding the oocyte (Figure 1A). The specificity of the anti-ZP antibodies was confirmed by detection of heterologously expressed mZP1, mZP2, and mZP3 in both immunocytochemistry and Western blots (Supplementary Figure S1 and Figure 1C). On Western blots of solubilized ZPs isolated from mouse oocytes, the antibodies detected proteins with apparent molecular weights (M_*w*_) of about 150, 100, and 83 kDa for mZP1, mZP2, and mZP3, respectively (Figure 1B), similar to what has been shown previously (Bleil and Wassarman, 1980; Wassarman, 1988; Thaler and Cardullo, 1996). Treatment with PNGase F decreased the M_*w*_, demonstrating that glycosylation of ZP proteins was preserved during isolation (Figure 1B).

We analyzed the action of ZP proteins on mouse sperm using a stopped-flow apparatus and fluorescent probes for Ca^2+^ and pH_*i*_. Mixing of capacitated sperm (incubated for 90 min in buffer containing 3 mg/ml BSA and 25 mM ${\text{HCO}}_{3}^{-}$ at pH 7.4) with ZP proteins evoked a rapid Ca^2+^ increase that reached a plateau within 30–40 s (Figure 1D); the control signal evoked by mixing with buffer alone was subtracted, setting the control-signal level consistently to ΔF/F_0_ (%) = 0. Simultaneous alkalization and depolarization of sperm by mixing with buffer adjusted to pH 8.6 and containing a high K^+^ and a low Na^+^ concentration (dubbed K8.6 buffer) (Babcock and Pfeiffer, 1987) also evoked a rapid Ca^2+^ increase that reached a slightly higher [Ca^2+^]_*i*_ (Figure 1D). As a reference for the maximal signal amplitude at saturation of the Ca^2+^ indicator, we recorded the Ca^2+^ response evoked by the Ca^2+^ ionophore ionomycin (Figure 1D). ZP proteins, K8.6 buffer, and ionomycin also evoked Ca^2+^ responses in non-capacitated sperm (Figure 1E and Supplementary Figures S2a,b). Yet, relative to the reference signal evoked by ionomycin, the amplitudes of ZP- and K8.6-evoked Ca^2+^ response were enhanced upon capacitation (Figure 1E and Supplementary Figure S2b). To verify that the Ca^2+^ responses are carried by CatSper, we measured Ca^2+^ responses in wild-type and *CatSper1*-deficient mice. Indeed, the Ca^2+^ responses were abolished in sperm from *Catsper1* deficient-mice (*Catsper1-KO*, Figure 1F) (Xia and Ren, 2009).

Next, we studied ZP-induced pH_*i*_ responses. Mixing of capacitated sperm with ZP proteins evoked a rapid pH_*i*_ increase that reached a plateau after 20–30 s (Figure 1G). Mixing of sperm with the weak base NH_4_Cl as a positive control also evoked a rapid pH_*i*_ increase that reached a slightly higher pH_*i*_ (Figure 1G). The ZP- and NH_4_Cl-evoked pH_*i*_ responses were similar in wild-type and *Catsper1*-KO sperm (Figure 1H).

Altogether, these results confirm that ZP action on sperm involves an increase of pH_*i*_ (Arnoult et al., 1996b) and a Ca^2+^ influx via CatSper (Xia and Ren, 2009), and that capacitation enhances the Ca^2+^ response (Arnoult et al., 1999). Moreover, because the pH_*i*_ increase is preserved in *Catsper1*-KO mice, we conclude that the pH_*i*_ increase is not evoked by Ca^2+^ influx via CatSper. Instead, the ZP-induced pH_*i*_ increase might underlie the ZP-activation of CatSper.

### The ZP-Induced Alkalization Does Not Involve the Na^+^/H^+^ Exchanger sNHE

We aimed to unravel the molecular players underlying the ZP-evoked alkalization. Na^+^/H^+^ exchange via sNHE has been proposed to take part in the ZP-evoked alkalization (Chavez et al., 2014). In addition to sNHE, two members of the Na^+^/H^+^ antiporter (NHA) subfamily, NHA1 and NHA2 (encoded by the *Slc9b1* and *Slc9b2* genes, respectively), have been identified in mouse sperm (Liu et al., 2010; Chen et al., 2016). Thus, NHA1 and NHA2 are also candidates to mediate the ZP-induced alkalization by Na^+^/H^+^ exchange.

We first probed the role of Na^+^/H^+^ exchange in the ZP-induced alkalization using Na^+^ substitution and pharmacology. Indeed, the ZP-induced pH_*i*_ response was abolished by substitution of extracellular Na^+^ by NMDG (*N*-methyl-D-glucamine) or addition of EIPA, a commonly used non-selective inhibitor of Na^+^/H^+^ exchangers (Shi et al., 2017). The NH_4_Cl-induced pH_*i*_ response was, however, similar in the absence or presence of Na^+^ or EIPA (Figures 1I–K). These results confirm that the ZP-induced pH_*i*_ response depends on Na^+^/H^+^ exchange.

**FIGURE 2:**
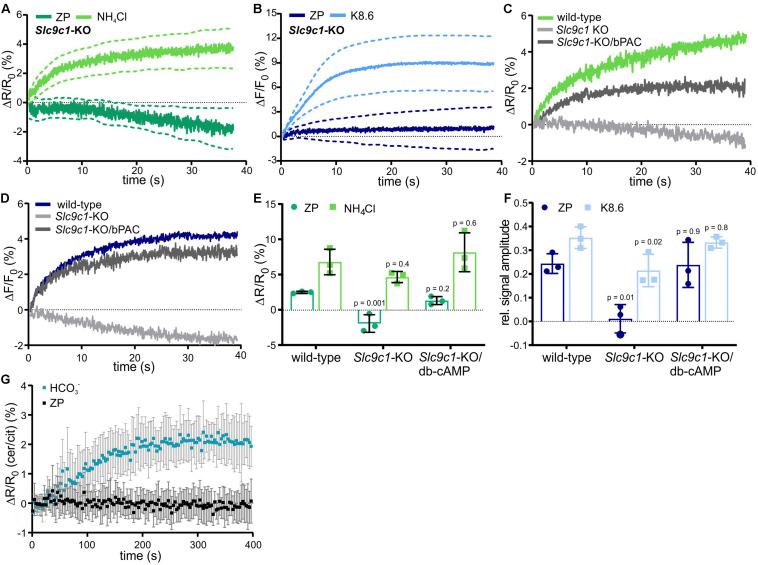
ZP-induced pH_*i*_ and Ca^2+^ responses in sperm lacking sNHE. Intracellular pH **(A)** and Ca^2+^ **(B)** responses in *Slc9c1*-KO sperm mean ± 95% confidence interval (*n* = 4). **(C)** Representative pH_*i*_ responses evoked by 1 ZP/μl in wild-type, *Slc9c1*-KO, and light-stimulated *Slc9c1*-KO/bPAC sperm. **(D)** Representative Ca^2+^ responses evoked by mixing with 1 ZP/μl in wild-type, *Slc9c1*-KO, and light-stimulated *Slc9c1*-KO/bPAC sperm. **(E)** Amplitudes of pH_*i*_ and **(F)** relative amplitudes of Ca^2+^ responses in wild-type, *Slc9c1*-KO, and *Slc9c1*-KO sperm pre-incubated with 5 mM db-cAMP (mean ± SD, *n* = 4). **(G)** Changes in the cerulean/citrine FRET ratio in mlCNBD-FRET sperm evoked by mixing with 50 mM NaHCO_3_ or 1 ZP/μl. An increase of the FRET ratio indicates an increase of free intracellular cAMP; mean ± SD, *n* = 5. Statistical comparison between multiple groups was performed using one-way ANOVA with Dunnett’s correction; *p*-values are indicated.

We examined if ZP-induced Na^+^/H^+^ exchange is mediated by sNHE using *Slc9c1* knockout-mice (*Slc9c1*-KO) (Wang et al., 2003). In *Slc9c1*-KO sperm, the ZP-induced pH_*i*_ and Ca^2+^ responses were abolished, whereas the pH_*i*_ and Ca^2+^ response evoked by NH_4_Cl and K8.6, respectively, was preserved (Figures 2A,B). However, sNHE interacts with the soluble adenylyl cyclase sAC, encoded by *Adcy10* (Wang et al., 2007), which constitutes the principal source of cAMP in mammalian sperm (Esposito et al., 2004; Hess et al., 2005; Xie et al., 2006); *Slc9c1*-KO sperm lack functional sAC and, therefore, cAMP synthesis (Wang et al., 2007). We wondered whether the failure of ZP proteins to increase [Ca^2+^]_*i*_ and pH_*i*_ in *Slc9c1*-KO is due to the lack of sNHE, sAC, or both. To test this, we used optogenetics and the membrane-permeable cAMP analog db-cAMP to rescue intracellular cAMP levels. Transgenic expression of the photoactivated adenylyl cyclase bPAC in *Slc9c1*-KO sperm provides a tool to stimulate cAMP synthesis in a light-dependent manner (Jansen et al., 2015). Light-stimulated cAMP synthesis in *Slc9c1*-KO/bPAC sperm or incubation of *Slc9c1*-KO sperm in db-cAMP both restored the ZP-induced pH_*i*_ and Ca^2+^ response (Figures 2C–F). Thus, the Na^+^/H^+^ exchange stimulated by ZP proteins does not require sNHE, but rather cAMP synthesis by sAC. Using sperm that express the FRET-based cAMP biosensor mlCNBD-FRET (Mukherjee et al., 2016), we investigated whether ZP proteins enhance sAC activity and, thereby, control intracellular cAMP synthesis. Mixing of mlCNBD-FRET sperm with ZP proteins did not increase cAMP levels, whereas activation of sAC by using 25 mM NaHCO_3_ as a control evoked a pronounced cAMP increase (Figure 2G), demonstrating that ZP proteins do not control cAMP levels in sperm. Altogether, we conclude that the ZP-induced Na^+^/H^+^ exchange is not mediated by sNHE, but requires cAMP.

### The ZP-Induced Alkalization Involves the Na^+^/H^+^ Exchanger NHA1

**FIGURE 3:**
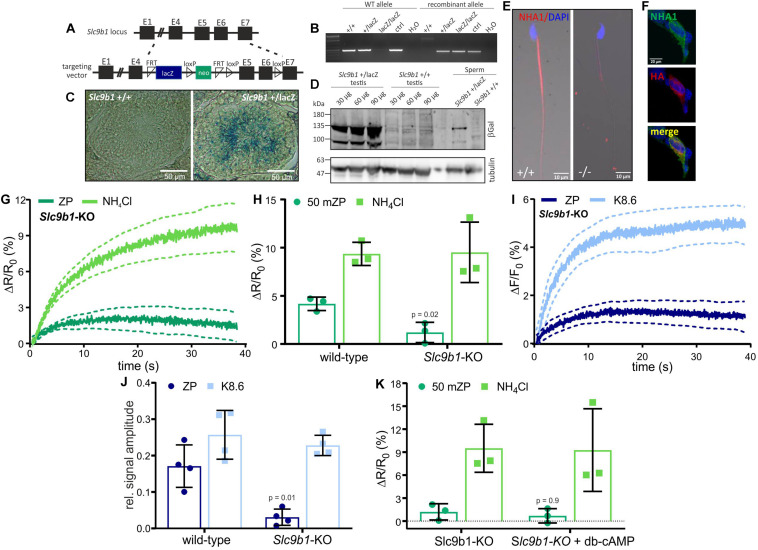
ZP-induced pH_*i*_ and Ca^2+^ response in sperm lacking NHA1. **(A)** Targeting strategy to delete the *Slc9b1* gene to generate *Slc9b1*-KO mice. **(B)** Genotyping PCR of wild-type (+/+), heterozygous (+ /lacZ), and homozygous (lacZ/lacZ) knockout mice. **(C)** X-gal staining of *Slc9b1*^+/+^ and *Slc9b1*^+/lacZ^ testis section; scale bar: 50 μm. **(D)** Western blot of testis and sperm lysates from Slc9b1^+/+^ and Slc9b1^+/lacZ^ mice, probed with anti-beta-galactosidase and anti-beta tubulin antibodies. **(E)** Immunostaining of wild-type and *Slc9b1*-KO sperm with an anti-NHA1 antibody; scale bar: 10 μm. **(F)** HEK293T cells heterologously-expressing NHA1, stained with an anti-NHA1 (green) and an anti-HA-tag antibody (red), co-localization shown in yellow, scale bar = 20 μm. **(G)** pH_*i*_ responses evoked by mixing with 1 ZP/μl or 10 mM NH_4_Cl in *Slc9b1*-KO mouse sperm; average ± 95% confidence interval (*n* = 3). **(H)** Amplitude of pH_*i*_ responses in wild-type or *Slc9b1*-KO mouse sperm; mean ± SD (*n* = 3). **(I)** Ca^2+^ responses evoked by mixing with 1 ZP/μl or K8.6 in *Slc9b1*-KO mouse sperm; average ± 95% confidence interval (*n* = 4). **(J)** Relative amplitude of Ca^2+^ responses evoked by mixing with 1 ZP/μl or K8.6 in wild-type or *Slc9b1*-KO mouse sperm; mean ± SD (*n* = 4). **(K)** Amplitude of pH_*i*_ responses in *Slc9b1*-KO sperm in the absence and presence of 5 mM db-cAMP; mean ± SD (*n* = 3). Statistical significance between two groups was determined using two-tailed, unpaired *t*-test with Welch’s correction; *p*-values are indicated.

NHA1, a member of the Na^+^/H^+^ antiporter subfamily encoded by the *Slc9b1* gene, was identified in the flagellum of mouse sperm (Liu et al., 2010; Chen et al., 2016). *Slc9b1*-KO sperm suffer from impaired motility, resulting in male subfertility (Chen et al., 2016). To investigate the role of NHA1 in the ZP-induced alkalization, we generated *Slc9b1*-KO mice. *Slc9b1*-KO mice were born in Mendelian ratios from heterozygous matings, and the mice were viable without any gross phenotype. The recombinant *Slc9b1* locus contains the *lacZ* gene, expressing beta-galactosidase under the control of the *Slc9b1* promoter (Figure 3A). Gene targeting was verified by PCR (Figure 3B). Labeling of beta-galactosidase in wild-type and heterozygous *Slc9b1*^+/lacZ^ testis sections and on Western blots of *Slc9b1*^+/lacZ^ testis and sperm lysates demonstrated that the *Slc9b1* gene is expressed in developing sperm (Figures 3C,D). Protein mass spectrometry identified NHA1 in wild-type sperm lysates (Supplementary Table S1). Furthermore, an anti-NHA1 antibody labeled the flagellum of wild-type, but not of *Slc9b1*-KO sperm (Figure 3E) and, as control, HEK293 cells heterologously expressing mouse NHA1 (Figure 3F). Altogether, these results confirm that NHA1 is expressed in wild-type, but not in *Slc9b1*-KO mouse sperm (Liu et al., 2010; Chen et al., 2016).

We investigated the action of ZP proteins on *Slc9b1*-KO sperm. The ZP-induced pH_*i*_ and Ca^2+^ responses were strongly attenuated, whereas the pH_*i*_ and Ca^2+^ responses evoked by NH_4_Cl or K8.6, respectively, were similar in wild-type and *Slc9b1*-KO (Figures 3G–J). These results suggest that the ZP-induced alkalization depends predominantly on Na^+^/H^+^ exchange via NHA1. However, genetic ablation of NHA1 reportedly affects sAC expression and, thereby, intracellular cAMP levels (Chen et al., 2016). Thus, the phenotype of *Slc9b1*-KO sperm might be caused by impaired or abolished cAMP synthesis, resembling the *Slc9c1*-KO phenotype (Wang et al., 2003). Yet, in contrast to *Slc9c1*-KO sperm, db-cAMP did not restore the ZP-induced pH_*i*_ response in *Slc9b1*-KO sperm (Figure 3K). These results indicate that lack of NHA1, rather than impaired cAMP synthesis, underlies the attenuated ZP-induced pH_*i*_ response in *Slc9b1*-KO sperm. We surmise that the residual Na^+^/H^+^ exchange in *Slc9b1*-KO mice is either mediated by NHA2, by another unknown Na^+^/H^+^ exchanger, or a combination of both.

### Genetic Ablation of NHA1 Affects Sperm Motility

Considering that the ZP-induced alkalization is predominantly mediated by NHA1, we analyzed the phenotype of *Slc9b1*-KO mice in more detail. In line with previous results, the fertility of *Slc9b1*-KO males was severely impaired (Chen et al., 2016): only 2 out of 21 matings (*Slc9b1*-KO males with wild-type females) produced offspring (Supplementary Table S2). The testis and epididymis weight and average sperm count were similar in wild-type and *Slc9b1*-KO mice (Supplementary Table S2), yet *Slc9b1*-KO sperm largely failed to fertilize oocytes *in vitro* (Supplementary Table S2), which might be attributed to the severely impaired ZP-induced Ca^2+^ and pH_*i*_ signaling. In previous studies, the number of sperm cells that were motile was significantly reduced in *Slc9b1*-KO mice (Chen et al., 2016). We observed a similar phenotype: only 55 ± 5% of *Slc9b1*-KO sperm showed progressive motility compared to 86 ± 5% of wild-type sperm. The decrease in motility has been attributed to a reduced sAC protein expression level (Chen et al., 2016). Thus, we investigated sAC function in wild-type versus *Slc9b1*-KO sperm. To this end, we studied the flagellar beat frequency (Hansen et al., 2018), which is controlled by sAC: activation of sAC by NaHCO_3_ rapidly increases intracellular cAMP levels and the flagellar beat frequency. In sperm that lack sAC, cAMP synthesis and the action of NaHCO_3_ is abolished, rendering the sperm immotile (Wennemuth et al., 2003; Esposito et al., 2004; Hess et al., 2005; Xie et al., 2006; Carlson et al., 2007). To investigate whether sAC dysfunction underlies the defect in sperm motility in *Slc9b1*-KO sperm, we compared the basal flagellar beat frequency, determined at about 60 μm distance from the center of the sperm head, between wild-type and *Slc9b1*-KO sperm. Under basal conditions, the beat frequency was similar (WT: 11 ± 3 and KO: 13 ± 3 Hz, *n* ≥ 13), and stimulation with 25 mM NaHCO_3_ increased the frequency to a similar extent (WT: 20 ± 6% and KO: 22 ± 9%, *n* ≥ 13). Thus, the lack of NHA1 does not impair the sAC-control of flagellar beat frequency, suggesting that cAMP synthesis is not impaired.

**FIGURE 4:**
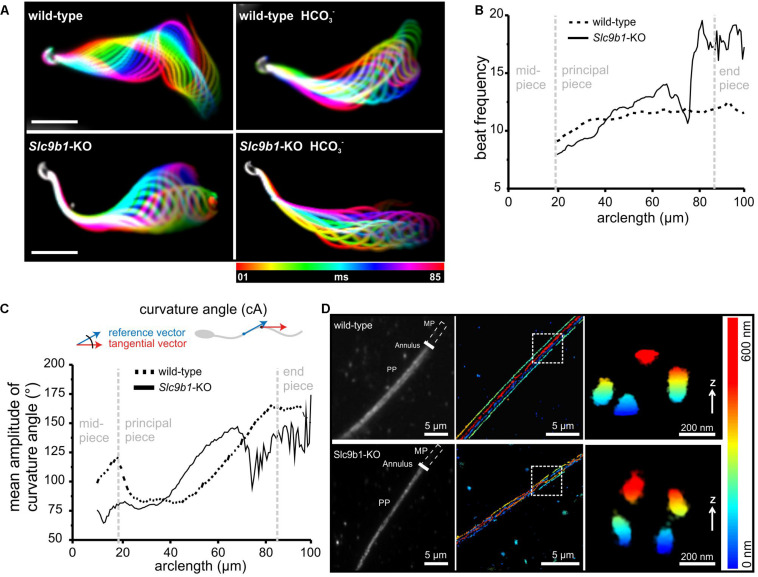
Flagellar beating pattern and ultrastructure in sperm lacking NHA1. **(A)** Flagellar waveform of wild-type and *Slc9b1*-KO sperm before and after stimulation with 25 mM NaHCO_3_. Superimposed color-coded frames taken every 5 ms, illustrating one flagellar beat cycle; scale bar: 30 μm. **(B)** Mean flagellar beat frequency along the flagellum of wild-type and *Slc9b1*-KO sperm (*n* = 5). **(C)** Mean amplitude of the curvature angle along the flagellum of wild-type and *Slc9b1*-KO sperm (*n* = 5). **(D)** Left: Epifluorescence image of wild-type and Slc9b1-KO sperm labeled with an anti-CatSper1 antibody. Position of the midpiece (MP), the annulus, and the principal piece (PP) is indicated. Middle: 3D-STORM image of the principal piece of wild-type and *Slc9b1*-KO sperm in x-y projection and color-coded for z; scale bars are indicated. Position of Z cross-section is indicated by white square. Right: Z cross-section of principal piece showing the four CatSper-organized signaling domains along the flagellum of wild-type and *Slc9b1*-KO sperm.

When analyzing the flagellar beat in detail, we noticed that the beat frequency along the flagellum was not uniform in *Slc9b1*-KO (Figures 4A,B and Supplementary Movies S1–S2). At ≤ 80 μm distance from the sperm head, the flagellar beat frequency was similar in *Slc9b1*-KO and wild-type sperm, whereas at > 80 μm, the frequency was considerably faster in *Slc9b1*-KO sperm (Figure 4B). Strikingly, *Slc9b1*-KO sperm displayed a stiff midpiece, which prevented to reliably determine the beat frequency at the first 20 μm of the flagellum. To describe this defect in quantitative terms, we compared the amplitudes of the curvature angle along the flagellum as a measure for the beat amplitude (Figure 4C) (Hansen et al., 2018). In the midpiece, the amplitude was lower in *Slc9b1*-KO sperm compared to wild-type sperm, reflecting the restricted movement (Figure 4C). Farther along the flagellum, the amplitude was similar between wild-type and *Slc9b1*-KO sperm (Figure 4C).

A stiff midpiece has also been observed in sperm lacking calcineurin or the CatSper-channel subunit CatSper ζ (Miyata et al., 2015; Chung et al., 2017). The CatSper-channel complex forms Ca^2+^ signaling domains along the flagellum that are organized in four longitudinal columns (Chung et al., 2014). Loss of the channel complex disrupts this organization (Chung et al., 2014, 2017). To examine whether a defect in the Ca^2+^ signaling domains might underlie the stiff midpiece in *Slc9b1*-KO sperm, we used super-resolution microscopy (3D-STORM). In wild-type and *Slc9b1*-KO sperm, an anti-CatSper1 antibody labeled four distinct columns aligned longitudinally along the flagellum, as previously described for wild-type sperm using the same antibody (Chung et al., 2014), demonstrating that the Ca^2+^-signaling domains are preserved in *Slc9b1*-KO sperm (Figure 4D). Furthermore, the density distribution of CatSper1 labeling along the flagellum was not different between wild-type and *Slc9b1*-KO sperm (Supplementary Figure S3). Thus, the molecular mechanism underlying the motility defect of *Slc9b1*-KO sperm remains unclear, but we propose that a combination of defective ZP signaling and altered flagellar beat pattern in *Slc9b1*-KO sperm underlies the sub-fertility of male *Slc9b1*-KO mice.

### ZP-Induced Signaling Requires a Sufficiently Negative Membrane Potential

**FIGURE 5:**
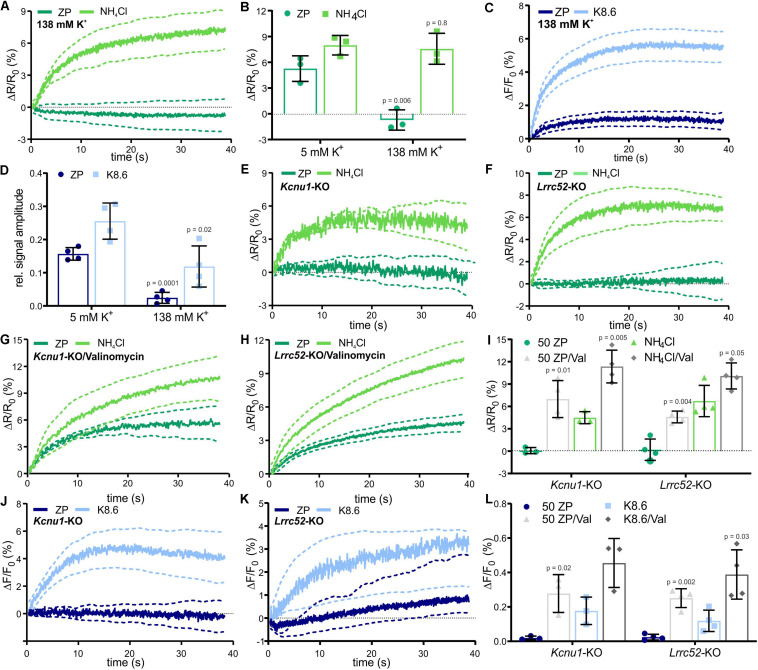
ZP-evoked pH_*i*_ and Ca^2+^ responses in mouse sperm lacking Slo3 or its auxiliary subunit Lrrc52. **(A)** pH_*i*_ responses evoked by mixing with 1 ZP/μl or 10 mM NH_4_Cl in sperm bathed in 138 mM K^+^ buffer; average ± 95% confidence interval (*n* = 3). **(B)** Amplitude of pH_*i*_ responses evoked by mixing with 1 ZP/μl or 10 mM NH_4_Cl in sperm bathed in 5 mM or 138 mM K^+^ buffer; mean ± SD (*n* = 3). **(C)** Ca^2+^ responses evoked by mixing with 1 ZP/μl or K8.6 in sperm bathed in 138 mM K^+^ buffer; average ± 95% confidence interval (*n* = 3). **(D)** Relative amplitude of Ca^2+^ responses evoked by mixing with 1 ZP/μl or K8.6 in sperm bathed in 5 or 138 mM K^+^ buffer; mean ± SD (*n* = 3). **(E,F)** pH_*i*_ responses evoked by mixing with 1 ZP/μl or 10 mM NH_4_Cl in Kcnu1-KO **(E)** and Lrrc52-KO **(F)** sperm; average ± 95% confidence interval (*n* = 3). **(G,H)** pH_*i*_ responses evoked by mixing with 1 ZP/μl or 10 mM NH_4_Cl in Kcnu1-KO **(G)** and Lrrc52-KO **(H)** sperm bathed in 2 μM valinomycin; average ± 95% confidence interval (*n* = 3). **(I)** Amplitude of pH_*i*_ responses evoked by mixing with 1 ZP/μl or 10 mM NH_4_Cl in Kcnu1-KO and Lrrc52-KO sperm in the absence and presence of 2 μM valinomycin; mean ± SD (*n* = 3). **(J,K)** Ca^2+^ responses evoked by mixing with 1 ZP/μl and K8.6 in Kcnu1-KO **(J)** and Lrrc52-KO **(K)** sperm; average ± 95% confidence interval (*n* = 3). **(L)** Relative amplitude of Ca^2+^ evoked by mixing with 1 ZP/μl or K8.6 in Kcnu1-KO and Lrrc52-KO sperm in the absence and presence of 2 μM valinomycin; mean ± SD (*n* = 3). Statistical significance between two groups was determined using two-tailed, unpaired *t*-test with Welch’s correction; *p*-values are indicated.

Although it is unknown how ZP proteins activate Na^+^/H^+^ exchange via NHA1, the membrane potential seems to be an important factor (Zeng et al., 1995; Arnoult et al., 1999; De La Vega-Beltran et al., 2012; Chavez et al., 2014). Thus, we tested the role of the V_*m*_ in ZP signaling. In wild-type sperm, that were depolarized by incubation in high extracellular potassium ([K^+^]_*o*_ = 138 mM), the ZP-induced pH_*i*_ and Ca^2+^ responses were abolished, whereas the pH_*i*_ and Ca^2+^ responses evoked by NH_4_Cl or K8.6, respectively, were preserved (Figures 5A–D). This suggests that the ZP-induced pH_*i*_ increase requires a more negative V_*m*_. Slo3 and its auxiliary subunit Lrrc52 form the principal K^+^ channel in mouse (Santi et al., 2010; Yang et al., 2011; Zeng et al., 2011, 2015) and human sperm (Brenker et al., 2014). We studied the action of ZP proteins on *Kcnu1*- and *Lrrc52*-KO sperm, which both feature a depolarized V_*m*_ (Santi et al., 2010; Yang et al., 2011; Zeng et al., 2011, 2015). In *Kcnu1*-KO and *Lrrc52*-KO sperm, the ZP-evoked pH_*i*_ and Ca^2+^ responses were abolished, whereas the pH_*i*_ and Ca^2+^ responses evoked by NH_4_Cl and K8.6, respectively, were preserved (Figures 5E,F,I–L). Remarkably, hyperpolarizing *Kcnu1*- and *Lrrc52*-KO sperm using the K^+^ ionophore valinomycin (Santi et al., 2010; Chavez et al., 2014) restored the ZP-induced pH_*i*_ and Ca^2+^ responses (Figures 5G–I,L), supporting the notion that ZP signaling requires a sufficiently negative V_*m*_. Altogether, these results corroborate that a negative V_*m*_, set by Slo3, enables the ZP-induced pH_*i*_ increase and, thereby, ZP-induced Ca^2+^ influx via CatSper. However, the mechanism underlying the V_*m*_-control of NHA1 remains to be elucidated.

## Discussion

**FIGURE 6:**
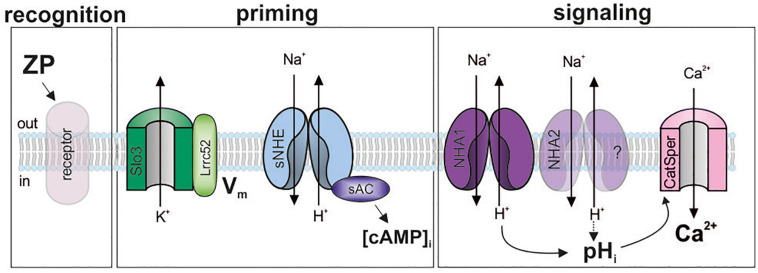
Model of the ZP-signaling pathway in mouse sperm. ZP glycoproteins bind to a yet unknown receptor (recognition), which initiates a signaling cascade. This cascade requires a hyperpolarized membrane potential (V_*m*_), set by the Slo3/Lrrc52-channel complex, and cAMP synthesis by sAC (priming). Thereby, a pH_*i*_ response, resting on Na^+^/H^+^ exchange by NHA1, and probably NHA2, is enabled that promotes Ca^2+^ influx through CatSper (signaling). Shaded objects indicate that the signaling mechanism and function of the molecules has not been established yet.

The function of mammalian sperm is controlled by external cues that engage various signaling molecules. How these molecules are integrated into signaling pathways is not well-understood. Here, we show that the synthesis of cAMP and a sufficiently negative membrane potential prime mouse sperm to transduce binding of ZP proteins into rapid H^+^ and Ca^2+^ signaling events (Figure 6). This ZP-induced Ca^2+^ increase might be involved in the control of swimming behavior and acrosomal exocytosis.

The CatSper channel is the principal pathway for Ca^2+^ entry into mammalian sperm (Quill et al., 2001; Ren et al., 2001; Kirichok et al., 2006; Lishko and Kirichok, 2010). We propose that the action of ZP proteins on CatSper is indirect, mediated by a pH_*i*_-signaling pathway (Figure 6): any experimental condition that abolishes the ZP-induced pH_*i*_ increase abolishes the Ca^2+^ influx, and vice versa, conditions that restore the pH_*i*_ response also restore the Ca^2+^ influx. This result is consistent with earlier reports, demonstrating a ZP-evoked increase of pH_*i*_ and [Ca^2+^]_*i*_ in mouse sperm (Bailey and Storey, 1994; Murase and Roldan, 1996). In patch-clamp experiments, ZP proteins did not enhance monovalent CatSper currents recorded from sperm isolated from the corpus of the ductus epididymis (Xia and Ren, 2009). Yet, sperm from this region of the epididymis are unable to undergo capacitation (Yanagimachi, 1994), which potentiates the Ca^2+^ response (see Figure 1D). Thus, the use of non-capacitated sperm might have hampered the detection of a direct ZP protein action on CatSper. Nevertheless, these results support our model that in mouse sperm, ZP proteins activate CatSper via intracellular alkalization (Figure 6). The ZP-induced pH_*i*_ increase rests on Na^+^/H^+^ exchange that is predominantly mediated by NHA1 (Figure 6). The residual pH_*i*_ response in *Slc9b1*-KO might be carried by NHA2; future work needs to address this question using *Scl9b1/2* knockout mice. The pH_*i*_ response is only observed at the negative V_*m*_ set by Slo3 (Santi et al., 2010; Zeng et al., 2011, 2015). It remains to be elucidated how the V_*m*_ dependence is integrated into the ZP-signaling pathway. In mouse sperm, K^+^ currents and, thus, V_*m*_ are strongly controlled by pH_*i*_ (Navarro et al., 2007; Zeng et al., 2011, 2013). The control of the ZP-induced pH_*i*_ response by V_*m*_, the control of V_*m*_ by Ca^2+^ and pH_*i*_, and the interplay of CatSper and Slo3 during ZP signaling deserve further studies.

We identified cAMP as a key player in the ZP-signaling pathway in mouse sperm: basal cAMP synthesis by sAC is required for the pH_*i*_ response and ensuing CatSper activation. sNHE, which might form a physical or functional complex with sAC, is however not responsible for the ZP-induced alkalization. It has been proposed that NHA1 and NHA2 control sAC expression and, thereby, cAMP synthesis and motility (Chen et al., 2016). We also find *Slc9b1*-KO males are infertile and feature a significantly reduced number of motile sperm. However, we show that sAC function is unaffected in NHA1-KO sperm as the control of the flagellar beat frequency by ${\text{HCO}}_{3}^{-}$ remains unchanged. Thus, the pathomechanism underlying the reduced number of motile sperm in *Slc9b1*-KO males remains to be elucidated. Yet, the loss of NHA1 alters the flagellar beat pattern along the flagellum with the midpiece being stiff and the rest of the flagellum being more flexible. Although the ZP-induced alkalization via NHA1 activates CatSper, the exchanger is not required for the organization of CatSper in quadrilateral columns. Future studies are required to elucidate whether NHA1 is part of these columns to form nanodomains of pH and Ca^2+^ signaling. Whether the loss of NHA1 affects other downstream processes that control the flagellar beat pattern or if the axonemal structure/dynein function is altered is not known and warrants further studies.

Sperm must undergo the acrosome reaction to penetrate through the ZP. Across species, binding of sperm to the native ZP, to isolated ZPs, as well as to solubilized ZP proteins evokes the acrosome reaction in sperm (Florman and Storey, 1982; Bleil and Wassarman, 1983; Cherr et al., 1986; O’Rand and Fisher, 1987; Cross et al., 1988; Florman and First, 1988; Uto et al., 1988; Arnoult et al., 1996a; Schroer et al., 2000; Tollner et al., 2003; Gupta et al., 2012). This suggested that *in vivo*, sperm undergo the acrosome reaction primarily upon binding to the ZP (Hirohashi and Yanagimachi, 2018). Recent studies utilizied fluorescently-labeled sperm from transgenic mouse models to determine the acrosomal status and acrosome reaction in live mouse sperm upon fertilization *in vitro* and *ex vivo* within the oviduct. These experiments revealed that at least in mice, most sperm undergo the acrosomal exocytosis during their journey across the oviduct or within the cumulus cell-matrix rather than upon binding to the zona pellucida (Jin et al., 2011; Hino et al., 2016; La Spina et al., 2016; Muro et al., 2016). Moreover, acrosome-reacted mouse sperm seem to reach the surface of the oocyte more readily than acrosome-intact sperm (Hildebrand et al., 2010), and mouse sperm can bind to the ZP without undergoing the acrosome reaction (Baibakov et al., 2007). Thus, the significance of the acrosomal exocytosis and signaling events evoked by binding of sperm to the ZP for fertilization in mice or other mammalian species still remain to be elucidated.

In summary, our study provides new insights and at the same time, raises new questions about the action of ZP proteins on mammalian sperm. The identification of the ZP receptor(s) and binding site(s) on sperm is certainly essential to delineate the whole ZP-signaling pathway. However, this question has remained unanswered since the initial characterization of ZP-sperm interaction (Bleil and Wassarman, 1980). Our results present NHA1, sAC, and Slo3/Lrrc52 as new players in the sperm ZP- signaling pathway. This might be the basis for future approaches, unraveling the molecular mechanisms underlying infertility and the design of new contraceptives.

## Materials and Methods

### Nomenclature

For most of the proteins, we have used the alias in the text. The official nomenclature is indicated in Table 1.

### Generation of *Slc9b1*-KO Mice

*Slc9b1*-KO mice were generated by blastocyst injection of *Slc9b1* mutant embryonic stem (ES) cells (EPD0187_1_D11, EUCOMM) into albino C57BL/6Tyr females (Transgenic Service, LIMES institute, University of Bonn) and backcrossed to C57Bl/6N. The offspring was genotyped by PCR using *Slc9b1*-specific primers (wild-type allele: 507 bp using #1: 5′-TAGGTTGAGTTCTACTACAATG-3′, #2: 5′-TAGAGTCCATAGTGCAATGAG-3′; floxed allele: 673 bp using #1/2; lacZ allele: 427 bp using #1 and #3: 5′-AGTCTTCCTGTCCAGG-3′). Mice used in this study were 2–5 months of age. Animal experiments were in accordance with the relevant guidelines and regulations and approved by the local authorities (LANUV) AZ 84-02.04.2012.A192 [intern BCI_10]. Fertility was tested using timed matings (wild-type females mated with *Slc9b1*-KO males overnight and separated after plug check the following morning). All females were plug positive, but only 2/21 *Slc9b1*-KO males produced offspring.

### Transgenic and Knockout Mice

*Catsper1*-KO (Ren et al., 2001) and *Slc9c1*-KO mice (Wang et al., 2003) were provided by David Clapham (Janelia Research Campus, United States) and the Jackson laboratory, respectively. *Kcnu1*-KO and *Lrrc52*-KO mice were provided by Christopher Lingle (Yang et al., 2011; Zeng et al., 2015). Prm1-bPAC/*Slc9c1*-KO mice have been described before (Jansen et al., 2015).

### Sperm Preparation

Mouse sperm were isolated as previously described (Raju et al., 2015). In brief, sperm were isolated by incision of the cauda epididymis followed by a swim-out in modified TYH medium (in mM: 135 NaCl, 4.8 KCl, 2 CaCl_2_, 1.2 KH_2_PO_4_, 1 MgSO_4_, 5.6 glucose, 0.5 sodium pyruvate, 10 lactic acid, 10 HEPES, pH 7.4 adjusted at 37°C with NaOH). After 15–30 min swim-out at 37°C, sperm were collected and counted. For capacitation, sperm were incubated for 90 min in TYH containing 3 mg/ml BSA and 25 mM NaHCO_3_ substituting 25 mM of the NaCl; the pH was adjusted to 7.4. Measurements under depolarized membrane potential were performed in TYH with 135 mM KCl and 5 mM NaCl at pH 7.4. For experiments in the absence of extracellular Na^+^, Na^+^ was substituted by *N*-methyl-D-glucamine (M2004, Sigma-Aldrich) and the pH was adjusted with HCl. Valinomycin and db-cAMP were added after swim-out and were present throughout the experiment. Prm1-bPAC/*Slc9c1*-KO sperm were isolated under dim red light. Light-activation of Prm1-bPAC/*Slc9c1*-KO sperm was performed in a custom-made rack equipped with blue LEDs during sperm capacitation. Experiments were performed with capacitated sperm unless otherwise indicated. The cAMP analogs (db-cAMP) were already added during the capacitation phase for 90 min.

### Isolation of Mouse *Zona pellucidae*

For ZP isolation, wild-type female mice were super-ovulated by intraperitoneal injection of 10 I.U. hCG (human Chorionic Gonadotropin; ProSpec, Rehovot, Israel) 3 days before the experiment. 14 h before oocyte isolation, mice were injected with 10 I.U. PMSG (Pregnant Mare’s Serum Gonadotropin; ProSpec). Mice were killed by cervical dislocation and oviducts were dissected. Cumulus-enclosed oocytes were prepared from the oviducts in TYH buffer containing 300 μg/ml hyaluronidase (Sigma). After 15 min, cumulus-free oocytes were transferred into fresh buffer and washed twice. *Zonae pellucidae* and oocytes were separated by shear forces generated by expulsion from 50 nm pasteur pipettes. *Zona pellucidae* were counted, transferred into fresh buffer, diluted to a concentration of 1 ZP per ul, and solubilized by incubation at 75°C for 15 min (Thaler and Cardullo, 1996). Animal experiments were performed in accordance with the relevant guidelines and regulations and approved by the local authorities (LANUV) AZ84-02.05.40.13.127.

### Heterologous Expression of ZP Glycoproteins

The cDNA sequence of mZP1, mZP2, and mZP3 was amplified via PCR. A hexa-histidine tag was inserted upstream of the conserved furin cleavage site, an *Age*I restriction site was added to the 5′ end, and a Kpnl restriction site to the 3′ end by nested PCR. The PCR product was cloned into a pHLsec vector (kindly provided by Prof. Yvonne Jones, Wellcome Trust Center for Human Genetics, University of Oxford, United Kingdom) using *Age*I and Kpnl. pHLsec-mZP1, pHLsec-mZP2 and pHLsec-mZP3 were transiently transfected in HEK293T cells (ATCC-CRL-3216) using polyethyleneimine (Life Technologies, Carlsbad, United States).

### Western Blot Analysis

Total protein lysates were obtained by homogenizing the cells in lysis buffer (10 mM Tris/HCl, pH 7.6, 140 mM NaCl, 1 mM EDTA, 1% Triton X-100, mPIC protease inhibitor cocktail 1:500). Samples were incubated for 30 min on ice and centrifuged at 10,000 *g* for 5 min at 4°C. The protein concentration was determined by BCA assay. Prior to separation by SDS-PAGE, 50 isolated ZPs, cells, or tissue lysates were mixed with 4 × SDS loading buffer [200 mM Tris/HCl, pH 6.8, 8% SDS (w/v), 4% β-mercaptoethanol (vol/vol), 50% glycerol, 0.04% bromophenol blue] and heated for 5 min at 95°C. For Western blot analysis, proteins were transferred onto PVDF membranes (Merck Millipore, Billerica, United States), probed with antibodies, and analyzed using a chemiluminescence detection system. For deglycosylation, 50 ZPs were incubated for 1 h with PNGase-F (New England Biolabs) according to the manufacturer’s instructions.

Primary antibodies: anti-β-galactosidase (1:1000; Molecular Probes), anti-α-tubulin (1:5000; Sigma-Aldrich), antibodies against mouse ZP glycoproteins were isolated from the supernatant of hybridoma cell lines (mZP1: ATCC CRL-2464, mZP2: ATCC CRL-2463, mZP3: ATCC CRL-2463) and diluted 1:1000. Secondary antibody: goat-anti-rat, HRP conjugated (1:5000, Dianova), donkey-anti-rabbit, HRP conjugated (1:5000, Dianova).

### Immunocytochemistry

Sperm smeared on positively charged microscope slides were dried at room temperature (RT). For antigen retrieval, sperm were incubated in citrate buffer (10 mM sodium citrate, 0.05% Tween-20, pH 6) in a steamer at 99°C for 20 min and washed in PBS for 15 min at RT. Immunocytochemical analysis of CatSper1 expression was performed without antigen retrieval. Transiently transfected HEK293T cells, oocytes, or sperm were incubated in 4% PFA for 15 min and washed in PBS. To block unspecific binding sites, samples were incubated for 1 h with blocking buffer [0.5% Triton X-100 and 5% ChemiBLOCKER (Merck Millipore) in 0.1M phosphate buffer, pH 7.4]. The primary antibody was diluted in blocking buffer and incubated overnight. The fluorescent secondary antibody was diluted in blocking buffer containing 0.5 mg/ml DAPI (Life Technologies) and incubated for 1 h. Images were taken on a confocal microscope (FV1000; Olympus). Primary antibodies: anti-NHA1 (1:100; Biorbyt), anti-CatSper1 (1:250; Santa Cruz, sc-21180) (Chung et al., 2014), anti-His (1:100, Millipore); primary antibodies against mouse ZP glycoproteins were isolated from the supernatant of hybridoma cell lines (mZP1: ATCC CRL-2464, mZP2: ATCC CRL-2463, mZP3: ATCC CRL-2463) and diluted 1:100; anti-HA antibody (Roche). Secondary antibodies: donkey anti-rat Alexa488 (1:500; Dianova), donkey anti-rat A647 (1:1000; Life technologies), donkey anti-mouse Cy5 (1:500; Dianova).

### STORM Imaging and Analysis of Sperm Flagellar Proteins

STORM imaging experiments were performed in an imaging buffer (50 mM Tris, pH 8, 10 mM NaCl) with an oxygen scavenging system (0.5 mg/mL glucose oxidase, 40 μg/ml catalase, 10% glucose, and 10 mM 2-aminoethanethiol). 10.000–60.000 frames were acquired per data set using 647 nm excitation at 100 mW. A 405 nm laser was used to maintain an adequate number of localizations per frame. A cylindrical lens was introduced in the detection path for astigmatism 3D STORM acquisition. Perfect focus system from Nikon was used to minimize axial drift and a vibration isolation table was used to minimize lateral drift. STORM movies were analyzed as described previously using the Nikon software package based on a technology developed by Dr. Xiaowei Zhuang (Huang et al., 2008). Briefly, fluorescence peaks corresponding to individual molecules were identified in each frame and fit using least-squares or maximum-likelihood estimation with a two-dimensional Gaussian to determine the (x,y) position of each molecule. For 3D imaging, the ellipticity of the Gaussian fit was used to assign a z coordinate. Drift correction was applied using cross-correlation.

STORM images were rendered with each localization plotted as a Gaussian. Images were filtered to reject molecules with low photon number (below 500 photons). Molecules with aspect ratio higher than 1.5 for 2D and 2.5 for 3D datasets were rejected. Moreover, molecules that appear for > 10 consecutive frames were rejected. Non-specifically bound antibodies can give background in the STORM images, which appears as scattered localizations with low local densities. This background noise was removed by a local density filter. Low-density localizations were filtered out by removing a molecule if it was surrounded by fewer than 10 localizations in the 80 × 80 nm region.

### Beta-Galactosidase Staining of Testis Sections

Testis of adult male mice were isolated, punctured twice with a cannula and incubated overnight at RT in 4% PFA. After a single washing step in PBS for 10 min, testis were transferred into a 10% sucrose solution for 1 h and subsequently incubated overnight in 30% sucrose. On the next day, testis were embedded in Tissue TEK (Sakura Finetek) and stored at −80°C. The testis was sectioned in 16 μm thick cross-sections using a 2800 Frigocut-E cryostat (Reichert-Jung, Nußloch) at a knive temperature of −22°C. Sectioned tissue was washed three times for 5 min at RT with LacZ wash solution (100 mM NaH_2_PO_4_, 1.25 mM MgCl_2_, 2 mM EGTA, 0.1% w/v Deoxycholate, 0.2% w/v Nonidet P40, pH 7.4). Sections were incubated overnight at 37°C in LacZ substrate solution (100 mM NaH_2_PO_4_, 1.25 mM MgCl_2_, 2 mM EGTA, 50 mM K_3_[Fe(CN)_6_], 50 mM K_4_[Fe(CN)_6_], 8% w/v X-Gal, pH 7.4) and washed twice with H_2_O before being mounted on coverslips using Aqua-Poly/Mount (Polyscience).

### Measurement of Changes in Intracellular Ca^2+^ and pH in Mouse Sperm

Changes in [Ca^2+^]_*i*_ and pH_*i*_ in mouse sperm were recorded in a rapid-mixing device in the stopped-flow mode (SFM400; Bio-Logic, Claix, France) after loading with the fluorescent Ca^2+^ indicator Cal-520-AM (AAT Bioquest, Sunnyvale, United States) or the fluorescent pH indicator BCECF-AM (Thermo Fisher), respectively. Changes in [Ca^2+^]_*i*_ were measured as previously described (Strünker et al., 2011) with minor modifications. In brief, sperm were loaded with Cal-520-AM (5 μM) in the presence of Pluronic F-127 (0.02% v/v) at 37°C for 45 min. After incubation, excess dye was removed by three centrifugation steps (700 *g*, 7 min, RT). The pellet was resuspended in buffer and equilibrated for 5 min at 37°C. The sperm suspension (5 × 10^6^ sperm/ml) was rapidly mixed 1:1 (v/v, 100:100 μl) with the respective stimulant [ZP, K8.6, 2 μM ionomycin (Tocris)] at a flow rate of 0.5 ml/s. Fluorescence was excited by a SpectraX Light Engine (Lumencor, Beaverton, United States), whose intensity was modulated with a frequency of 10 kHz. The excitation light was passed through a BrightLine 475/28 nm filter (Semrock, Rochester, United States) onto the cuvette. Emission light was passed through a BrightLine 536/40 filter (Semrock) and recorded by photomultiplier modules (H10723-20; Hamamatsu Photonics). The signal was amplified and filtered through a lock-in amplifier (7230 DualPhase; Ametek, Paoli, United States). Data acquisition was performed with a data acquisition pad (PCI-6221; National Instruments, Austin, United States) and Bio-Kine software v. 4.49 (Bio-Logic). Ca^2+^ signal traces are depicted as the percent change in fluorescence (ΔF) with respect to the mean of the first three data points recorded immediately after mixing (F_0_). Mean ± 95% CI are shown to visualize the true range of the data. The control ΔF/F_0_ signal observed upon mixing with buffer (control) was subtracted from K8 6-, ZP-, or ionomycin-induced signals, setting the control-signal level to ΔF/F_0_ (%) = 0. The K8.6 solution (Babcock and Pfeiffer, 1987) contained (in mM: 4.8 NaCl, 138 KCl, 2 CaCl_2_, 1.2 KH_2_PO_4_, 1 MgSO_4_, 5.6 glucose, 0.5 sodium pyruvate, 10 lactic acid, 10 HEPES, pH 8.6, adjusted with KOH) to depolarize the V_*m*_ and simultaneously increase pH_*i*_ to activate CatSper. Bar graphs show the maximal amplitude of the ZP- or K8.6-evoked Ca^2+^ response (average of last three data points), normalized to the respective ionomycin-evoked Ca^2+^ response (average of last three data points) (relative signal amplitude). To measure pH changes in the stopped-flow mode, sperm were loaded with BCECF-AM (10 μM) at 37°C for 15 min. The pellet was resuspended in TYH and equilibrated for 5 min at 37°C. The excitation light was passed through a BrightLine 452/45 nm filter (Semrock) onto the cuvette. Emission light was passed in parallel through a BrightLine 494/20 filter and a BrightLine 540/10 filter (Semrock). pH signals are depicted as the percent change in fluorescence ratio (ΔR) of 494 nm/540 nm with respect to the mean of the first three data points recorded immediately after mixing (R_0_) when a stable fluorescence signal was observed. The ΔR/R_0_ signal evoked by mixing with buffer (control) was subtracted from ZP- or NH_4_Cl-induced signals. Bar graphs show the maximal amplitude of the ZP- or NH_4_Cl-evoked pH response (average of last three data points).

### *In vitro* Fertilization

Superovulation in females was induced as described above. HTF medium (EmbryoMax Human Tubal Fluid; Merck Millipore) was mixed 1:1 with mineral oil (Sigma-Aldrich) and equilibrated overnight at 37°C. Sperm were capacitated for 90 min in TYH medium supplemented as indicated above. On the day of preparation, 100 μl drops of HTF were covered with the medium/oil mixture and 10^5^ sperm were added to each drop. Cumulus-enclosed oocytes were prepared from the oviducts of superovulated females and added to the drops. After 4 h at 37°C and 5% CO_2_, oocytes were transferred to fresh HTF. The number of 2-cell stages was evaluated after 24 h.

### Sperm Motility Analysis

Freely beating sperm were observed in shallow perfusion chambers with 200 μm depth, which allowed to exchange the buffer during recordings. Sperm were tethered to the glass surface by lowering the BSA to 0.3 mg/ml. An inverted dark-field video microscope (IX71; Olympus) with a 10 x objective (UPlanFL, NA 0.4; Olympus) and an additional 1.6 x magnification lens (16x final amplification) was combined with a high-speed camera (Dimax; PCO). Dark-field videos were recorded with a frame rate of 200 Hz. The temperature was 37°C (Incubator; Life Imaging Services). The flagellar beat was analyzed using SpermQ (Hansen et al., 2018). SpermQ outputs the parameter curvature angle as a measure for flagellar bending. The curvature angle at a given point on the flagellum was determined by the angle between the tangential vector at the given point and the tangential vector at the point 10 μm proximal on the flagellum. The beat frequency at a given point on the flagellum was determined by the highest peak in the frequency spectrum obtained by Fast-Fourier-Transformation of the time-course of the parameter curvature angle at the given flagellar point. The amplitude of the curvature angle for a given point was determined as the absolute difference between the median of the five highest and the median of the five lowest curvature angle values in the entire time-course at the given point.

### Measuring cAMP Dynamics in Sperm

Population measurements using a spectrofluorometer (Quantamaster 40, PTI) were performed as previously described (Mukherjee et al., 2016) in PMMA cuvettes at 1 × 10^5^ cells/ml under constant stirring (Spinbar, Bel-art Products, Wayne, NJ, United States).

### Identification of NHA1 by Mass Spectrometry

Sperm were isolated from wild-type C57Bl/6 mice and subjected to mass spectrometry as described previously (Raju et al., 2015).

### Statistical Analysis

Statistical analyses for graphs shown in figures has been performed in GraphPad Prism. Statistical significance between two groups was determined using two-tailed, unpaired *t*-test with Welch’s correction, statistical significance between multiple groups was determined using one-way ANOVA with Dunnett’s correction. The respective details are indicated in the figure legends.

## Data Availability Statement

The raw data supporting the conclusions of this article will be made available by the authors, without undue reservation.

## Ethics Statement

The animal study was reviewed and approved by Landesamt für Natur, Umwelt und Verbraucherschutz Nordrhein-Westfalen (State Office for Nature, Environment and Consumer Protection North Rhine-Westphalia) [AZ 84-02.04.2012.A192 and AZ 84-02.05.40.13.127].

## Author Contributions

MB established and performed the pH_*i*_ and Ca^2+^ fluorimetry, prepared the native ZPs, and generated and analyzed the NHA1-KO. CS also performed pH_*i*_ and Ca^2+^ fluorimetry. HH performed STORM imaging. JJ and JH analyzed sperm motility. CT performed the mass spectrometry. PN performed genotyping of knockout mice. HF provided protocols for ZP isolation and analysis. HF, UK, CB, LJ, and LH analyzed and/or interpreted data, participated in drafting the manuscript, and revised the manuscript critically for important intellectual content. TS and DW conceived the project, designed and coordinated the experiments, analyzed and/or interpreted data, and wrote the manuscript. All authors contributed to the article and approved the submitted version.

## Conflict of Interest

The authors declare that the research was conducted in the absence of any commercial or financial relationships that could be construed as a potential conflict of interest.

## Supplementary Material

FIGURE S1 Validation of the specificity of anti-ZP antibodies using heterologously expressed protein. Immunostaining of HEK293T cells heterologously expressing mouse ZP glycoproteins with mZP isoform-specific (green) and His-tag antibodies (red), co-localization shown in yellow, **(a)** mZP1, **(b)** mZP2, **(c)** mZP3; scale bar = 10 μm.

FIGURE S2 Ca^2+^ responses in non-capacitated and capacitated mouse sperm. **(a)** Ca^2+^ responses evoked by mixing with 2 μM ionomycin in non-capacitated and capacitated sperm; average ± 95% confidence interval (*n* ≥ 18) **(b)** Ca^2+^ responses evoked by mixing with 1 ZP/μl, K8.6 or 2 μM ionomycin in non-capacitated and capacitated sperm; individual data points and mean ± SD (*n* ≥ 18).

FIGURE S3 Density distribution of CatSper domains along the flagella. **(a)** Normalized density profile of one CatSper domain along the arc length of wild-type (black) and *Slc9b1*-KO sperm flagella (red). **(b)** Fourier spectrum in dB of the data in **(a)**, wild-type (black) and *Slc9b1*-KO sperm flagella (red).

TABLE S1 NHA1 peptide sequences identified in total lysates from mouse sperm by mass spectrometry.

TABLE S2 Fertility parameters of male wild-type and *Slc9b1*-KO mice.

MOVIE S1 Flagellar motility of wild-type sperm before and after perfusion with 25 mM NaHCO_3_.

MOVIE S2 Flagellar motility of *Slc9b1*-KO sperm before and after perfusion with 25 mM NaHCO_3_.
