# Supplementary material for: Molecular Mechanism Underlying the Action of Zona-pellucida Glycoproteins on Mouse Sperm
Source: Front Cell Dev Biol. 2020 Aug 31;8:572735. doi: 10.3389/fcell.2020.572735 (PMC7487327; doi:10.3389/fcell.2020.572735)
Supplement: FIGURE S1 — Validation of the specificity of anti-ZP antibodies using heterologously expressed protein. Immunostaining of HEK293T cells heterologously expressing mouse ZP glycoproteins with mZP isoform-specific (green) and His-tag antibodies (red), co-localization shown in yellow, (a) mZP1, (b) mZP2, (c) mZP3; scale bar = 10 μm. [file Image_1.pdf]

Supplementary Figure 1

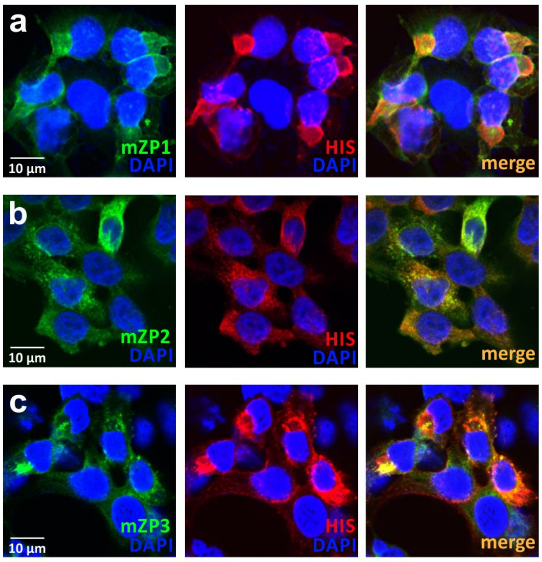

Supplementary figure 1: Validation of the specificity of anti-ZP antibodies using heterologously expressed protein. Immunostaining of HEK293T cells heterologously expressing mouse ZP glycoproteins with mZP isoform-specific (green) and His-tag antibodies (red), co-localization shown in yellow, (**a**) mZP1, (**b**) mZP2, (**c**) mZP3; scale bar = 10 µm.
