# Supplementary material for: Molecular Mechanism Underlying the Action of Zona-pellucida Glycoproteins on Mouse Sperm
Source: Front Cell Dev Biol. 2020 Aug 31;8:572735. doi: 10.3389/fcell.2020.572735 (PMC7487327; doi:10.3389/fcell.2020.572735)
Supplement: FIGURE S2 — Ca2+ responses in non-capacitated and capacitated mouse sperm. (a) Ca2+ responses evoked by mixing with 2 μM ionomycin in non-capacitated and capacitated sperm; average ± 95% confidence interval (n ≥ 18) (b) Ca2+ responses evoked by mixing with 1 ZP/μl, K8.6 or 2 μM ionomycin in non-capacitated and capacitated sperm; individual data points and mean ± SD (n ≥ 18). [file Image_2.pdf]

Supplementary Figure 2

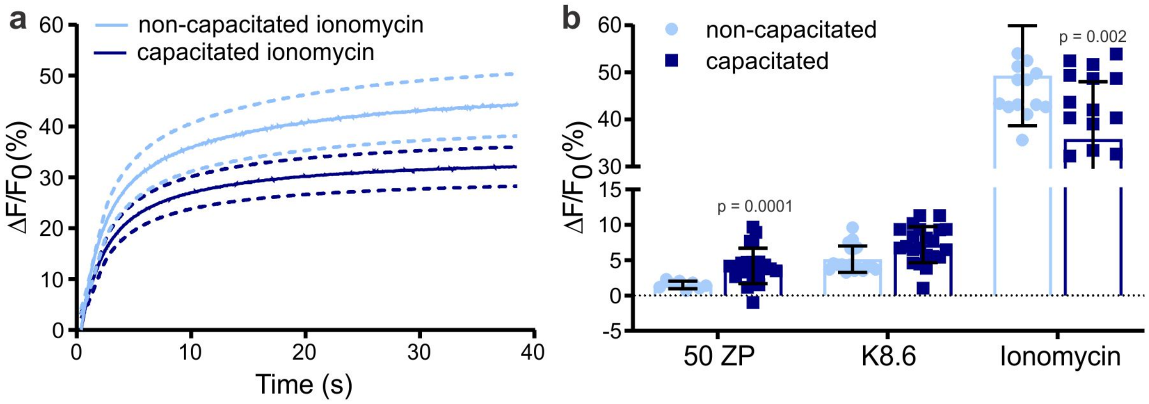

Supplementary figure 2: Ca^{2+} responses in non-capacitated and capacitated mouse sperm. (a) Ca^{2+} responses evoked by mixing with 2 μM ionomycin in non-capacitated and capacitated sperm; average ± 95 % confidence interval (n ≥ 18) **(b)** Ca^{2+} responses evoked by mixing with 1 ZP/μl, K8.6 or 2 μM ionomycin in non-capacitated and capacitated sperm; individual data points and mean ± SD (n ≥ 18).
