# Supplementary material for: Molecular Mechanism Underlying the Action of Zona-pellucida Glycoproteins on Mouse Sperm
Source: Front Cell Dev Biol. 2020 Aug 31;8:572735. doi: 10.3389/fcell.2020.572735 (PMC7487327; doi:10.3389/fcell.2020.572735)
Supplement: FIGURE S3 — Density distribution of CatSper domains along the flagella. (a) Normalized density profile of one CatSper domain along the arc length of wild-type (black) and Slc9b1-KO sperm flagella (red). (b) Fourier spectrum in dB of the data in (a), wild-type (black) and Slc9b1-KO sperm flagella (red). [file Image_3.pdf]

Supplementary Figure 3

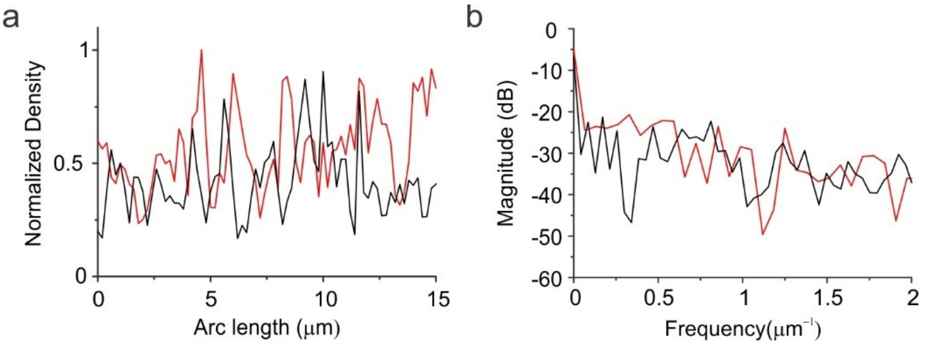

Supplementary figure 3. Density distribution of CatSper domains along the flagella.

(a) Normalized density profile of one CatSper domain along the arc length of wild-type (black) and *Slc9b1*-KO sperm flagella (red). **(b)** Fourier spectrum in dB of the data in **a**, wild-type (black) and *Slc9b1*-KO sperm flagella (red).
