# Supplementary material for: Molecular Mechanism Underlying the Action of Zona-pellucida Glycoproteins on Mouse Sperm
Source: Front Cell Dev Biol. 2020 Aug 31;8:572735. doi: 10.3389/fcell.2020.572735 (PMC7487327; doi:10.3389/fcell.2020.572735)
Supplement: TABLE S1 — NHA1 peptide sequences identified in total lysates from mouse sperm by mass spectrometry. [file Table_1.pdf]

Supplementary Table 1

NHA1 peptide sequences identified in total lysates from mouse sperm by mass spectrometry.

Peptide number	Peptide sequence	Amino acids
1	DPDSFHEETVEPKPELK	26-42
2	ETQTTEIER	88-96
3	IPVVPPLPPLIGMLLAGFTIR	168-188
4	NVPIIYEFVHIPTTWSSALR	189-208
5	IVANTWNVFQPLLFGLVGTEVSVESLESK	419-447
6	ATVQAVLGPLALETAR	492-507
7	ILEQSEVTFPLK	546-557
8	VELSNFHH	558-565
