# Supplementary material for: Molecular Mechanism Underlying the Action of Zona-pellucida Glycoproteins on Mouse Sperm
Source: Front Cell Dev Biol. 2020 Aug 31;8:572735. doi: 10.3389/fcell.2020.572735 (PMC7487327; doi:10.3389/fcell.2020.572735)
Supplement: TABLE S2 — Fertility parameters of male wild-type and Slc9b1-KO mice. [file Table_2.pdf]

Supplementary Table 2

Fertility parameters of male wild-type and *Slc9b1*-KO mice.

	**wild-type**	***Slc9b1*-KO**
ratio testis/body weight (mg/g)	3.73 ± 0.5 (n = 12)	3.65 ± 0.5 (n = 12)
ratio epididymis/body weight (mg/g)	0.74 ± 0.1 (n = 12)	0.83 ± 0.3 (n = 12)
sperm count (per ml)	1.6 ± 0.6 x 10^7 (n = 12)	1.5 ± 0.6 x 10^7 (n = 12)
mating with offspring	21/21 (100%)	2/21 (9.5%)
two-cell stage oocytes after IVF (%)	46 ± 7 (n = 5), 398 oocytes	2 ± 1 (n = 5), 443 oocytes
